# Atypical Adult-Onset IgA Vasculitis With Extremely Rare Complications Including Diffuse Alveolar Hemorrhage, Heart Failure, and Stroke

**DOI:** 10.7759/cureus.75607

**Published:** 2024-12-12

**Authors:** Mai Motojima, Yuki Tanabe, Fumihiko Makino, Hitoshi Suzuki, Shigeki Tomita, Shinichi Sasaki, Kazuhisa Takahashi

**Affiliations:** 1 Department of Respiratory Medicine, Juntendo University School of Medicine Graduate School of Medicine, Tokyo, JPN; 2 Department of Respiratory Medicine, Juntendo University Urayasu Hospital, Chiba, JPN; 3 Department of Nephrology, Juntendo University Faculty of Medicine, Tokyo, JPN; 4 Department of Nephrology, Juntendo University Urayasu Hospital, Chiba, JPN; 5 Department of Pathology, Juntendo University Urayasu Hospital, Chiba, JPN

**Keywords:** cardiac magnetic resonance, cmr, dah, diffuse alveolar hemorrhage, iga nephropathy, igav, iga vasculitis

## Abstract

IgA vasculitis (IgAV) generally occurs in young people and presents with a tetrad of symptoms: purpura, abdominal pain, arthralgia, and nephritis. However, it may have an atypical course without the typical tetrad. Diffuse alveolar hemorrhage (DAH), heart failure, and stroke are known complications of IgAV but are all very rare. We herein report a case of adult-onset IgAV that developed simultaneously with these rare complications without the typical tetrad. A 31-year-old man without any medical history presented with fever and blood-tinged sputum. Two months later, these symptoms worsened, and he was admitted to the hospital with DAH, nephritis, heart failure, and stroke. Initially, these symptoms were considered indicative of vasculitis syndrome, and he was finally diagnosed with IgAV based on the results of a renal biopsy. The treatment was successful with corticosteroids alone. IgAV should, therefore, be considered in the differential diagnosis when a patient presents with vasculitis syndrome, even with an atypical course.

## Introduction

Vasculitis syndrome is suspected in the presence of pulmonary renal syndrome (PRS), complicated by both diffuse alveolar hemorrhage (DAH) and nephritis, or when vascular disorders such as heart failure or stroke are present in addition to PRS [1].

IgA vasculitis (IgAV) is a vasculitic disorder characterized by the deposition of IgA1-dominant immune complexes in small vessels, typically resulting in a tetrad of symptoms, including purpura, abdominal pain, arthralgia, and nephritis [2]. However, it is very rare for IgAV to present with symptoms in the respiratory, cardiovascular, and central nervous systems, although some cases have been reported [3-5]. This case report describes a young man with an atypical course of IgAV, complicated by DAH, heart failure, and stroke, which was diagnosed through various tests, including renal biopsy. When a young person presents with severe clinical symptoms suggestive of vasculitis syndrome, IgAV should be considered as a differential diagnosis, even in the absence of the typical tetrad.

## Case presentation

A 31-year-old man without any past medical history presented with a fever and blood-tinged sputum. He reported these symptoms for two months, with recent worsening.

On admission, he had a fever of 38.3°C, blood pressure of 152/72 mmHg, a heart rate of 94 beats per minute, a respiratory rate of 20 breaths per minute, and an O2 saturation of 93% in room air. Physical examination revealed purpura on the lower extremities, although the patient had not initially reported this symptom. No other signs suggestive of vasculitis, such as gastrointestinal symptoms or joint pain, were noted.

Initial laboratory findings were significant for elevated creatinine (1.80 mg/dL; ref. 0.61-1.04 mg/dL), elevated C-reactive protein (1.7 mg/dL; ref. 0.0-0.3 mg/dL), and elevated N-terminal pro-brain natriuretic peptide (5975 pg/mL; ref. 0-125 pg/mL). Urinalysis indicated proteinuria (4+), microscopic hematuria (2+), and red blood cells (5-9/high power field). Granular, epithelial cells, and fatty casts were observed with elevated urinary β2-microglobulin (3561 μg/L; ref. 0-250 μg/L) and elevated urinary N-acetyl-beta-glucosaminidase (21.8 IU/L; ref. 0.7-11.2 IU/L). The autoimmune markers, including antinuclear antibodies, anti-neutrophil cytoplasmic antibodies (ANCAs), and anti-glomerular basement membrane (GBM) antibodies, were negative. Influenza and COVID-19 were ruled out based on negative rapid antigen tests, though comprehensive viral panels were not performed. Tuberculosis and other acid-fast bacterial infections were excluded through negative interferon-gamma release assay results and negative cultures (Table 1).

**Table 1:** Biochemical and biomarker test results on admission WBC: white blood cells, Hgb: hemoglobin, Plt: platelets, IgG: immunoglobulin G, IgA: immunoglobulin A, IgM: immunoglobulin M, NT-pro BNP: N-terminal pro-brain natriuretic peptide, MPO-ANCA: myeloperoxidase anti-neutrophil cytoplasmic antibody, PR3-ANCA: proteinase 3 anti-neutrophil cytoplasmic antibody, anti-GBM antibody: anti-glomerular basement membrane antibody, CRP: C-reactive protein, N. D.: not detected, RBC: red blood cells, hpf: high-power field

Laboratory data	Laboratory value	Reference range
Blood tests		
WBC	9.8	3.9-9.7 K/mcL
Hgb	16.4	13.4-17.1 g/dL
Plt	217	153-346 K/mcL
Blood urea nitrogen	21	8-22 mg/dL
Creatinine	1.8	0.61-1.04 mg/dL
Total protein	6.1	6.7-8.3 g/dL
Albumin	3.5	3.9-4.9 g/dL
IgG	835	870-1700 mg/dl
IgA	173	110-410 mg/dl
IgM	98	35-220 mg/dl
NT-pro-BNP	5975	0-125 pg/mL
Anti-nuclear antibody	<1:40	<1:40
Complement C3	104	65-135 mg/dL
Complement C4	29	13-35 mg/dL
MPO-ANCA	<1.0	<4.0 IU/ml
PR3-ANCA	<1.0	<4.0
Anti-GBM antibodies	<2.0	<3.0
CRP	1.7 mg/dL	<1.0 mg/dL
Urinalysis		
Proteinuria	4+	Negative
Protein, urine	386	0-20 mg/dL
Creatinine, urine	196.8	Not available
Protein/creatinine ratio	1.96	≤0.15 gPR/gCR
Microscopic hematuria	2+	Negative
Erythrocytes urinalysis	5 to 9/hpf	Not seen
β2-microglobulin	3561	0-250 μg/L
N-acetyl-beta-glucosaminidase	21.8	0.7-11.2 IU/L

**Figure 1:**
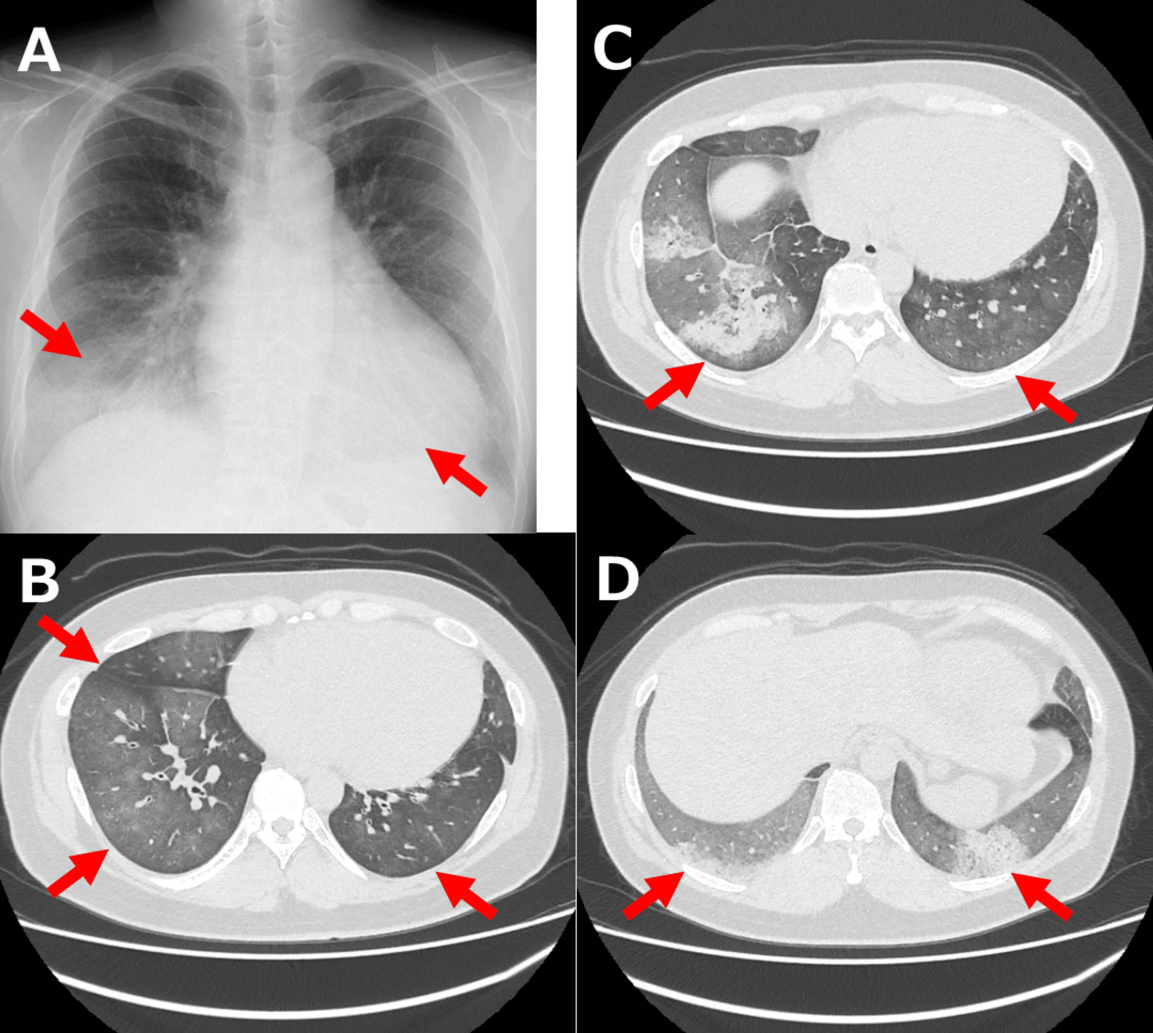
Chest imaging on admission A: Chest X-ray showing bilateral pulmonary infiltrates. B-D: Chest CT scan revealing diffuse ground-glass opacity with bilateral lower lobe predominance. CT: computed tomography

Chest X-ray showed bilateral pulmonary infiltrates, and a chest CT scan revealed diffuse ground-glass opacity with bilateral lower lobe predominance (Figure 1A-1D).

**Figure 2:**
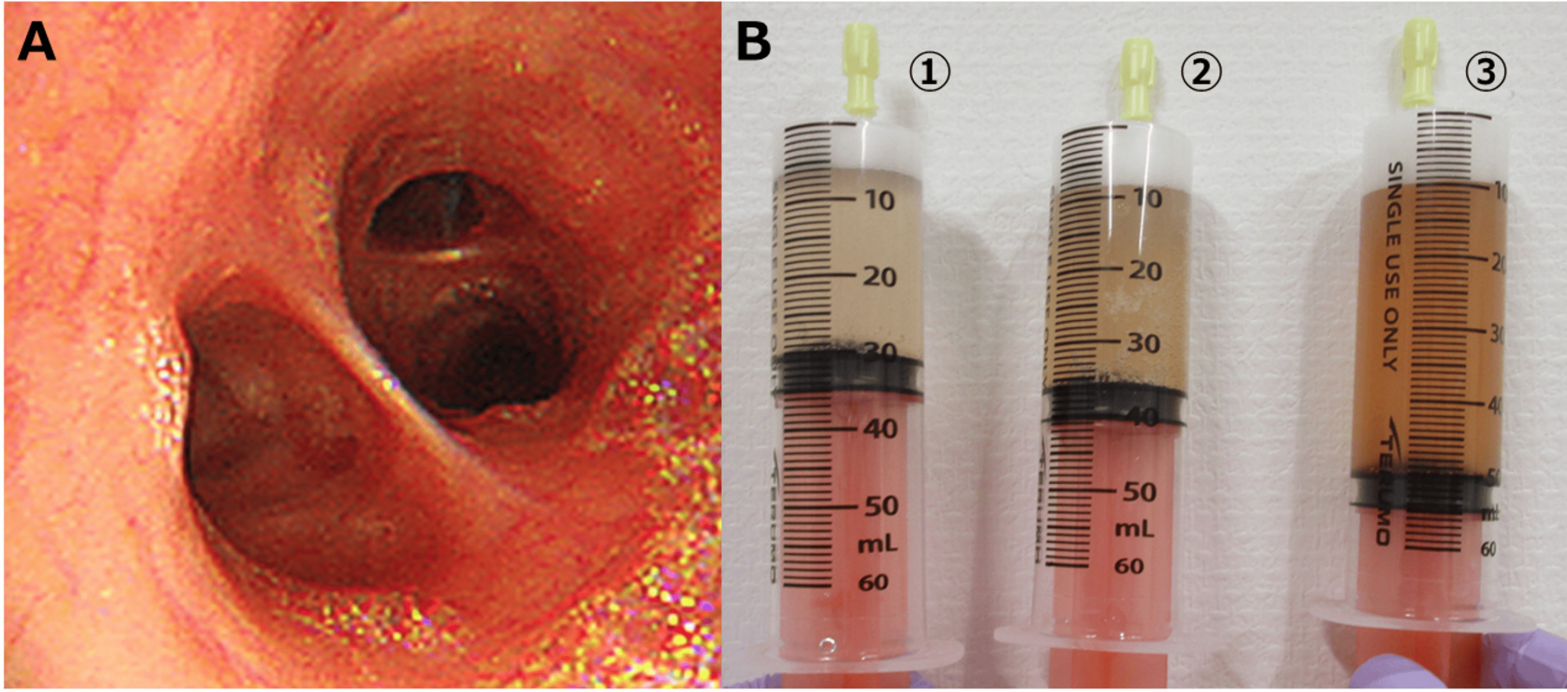
Bronchoscopic findings and BAL results A: Bronchoscopic examination revealed capillary engorgement and mucosal edema throughout the visible range from the trachea to the subsegmental bronchi without evidence of active bleeding. B: BAL was subsequently performed via the right B4. Sequential BAL samples from three consecutive procedures demonstrated a gradual increase in blood components and revealed 65% phagocytic macrophages, strongly indicating DAH. BAL: bronchoalveolar lavage, DAH: diffuse alveolar hemorrhage

Bronchoscopic examination revealed no evident active bleeding, but capillary dilation and airway edema were observed throughout the respiratory tract. Bronchoalveolar lavage (BAL) was subsequently performed via the right B4 and, on three consecutive occasions, demonstrated a gradual increase in blood components and revealed 65% phagocytic macrophages, strongly supporting the diagnosis of DAH (Figure 2A-2B).

A transthoracic echocardiogram (TTE) showed a decreased left ventricular ejection fraction (LVEF) of 20%. Due to concerns that these findings in the kidneys, lungs, and heart were secondary to vasculitis, the patient received 1000 mg methylprednisolone IV for three days, followed by oral prednisolone (PSL) of 70 mg/day (1 mg/kg/day). With this treatment, blood-tinged sputum and chest X-ray rapidly improved, but renal dysfunction persisted.

**Figure 3:**
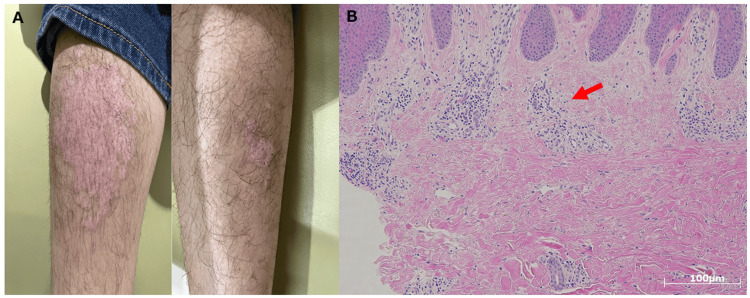
Clinical and histopathological findings of skin lesions A: Palpable purpura on the lower extremities. B: Histopathological examination of skin biopsy showing mild papillomatosis with hyperkeratosis and perivascular inflammatory cell infiltration predominantly composed of lymphocytes (H&E staining, scale bar = 100 μm). The red arrow indicates perivascular infiltration of inflammatory cells. H&E: hematoxylin and eosin

A skin biopsy was performed, which revealed inflammatory cell infiltration, including lymphocytes, although specific findings of leukocytoclastic vasculitis or IgA deposition were not observed (Figure 3A-3B). Notably, the absence of typical vasculitic changes or IgA deposition does not rule out IgAV, as these findings can be variable depending on the timing and location of the biopsy. The presence of inflammatory changes in the biopsy, combined with the clinical presentation of the rash, supports the possibility of a vasculitic process.

**Figure 4:**
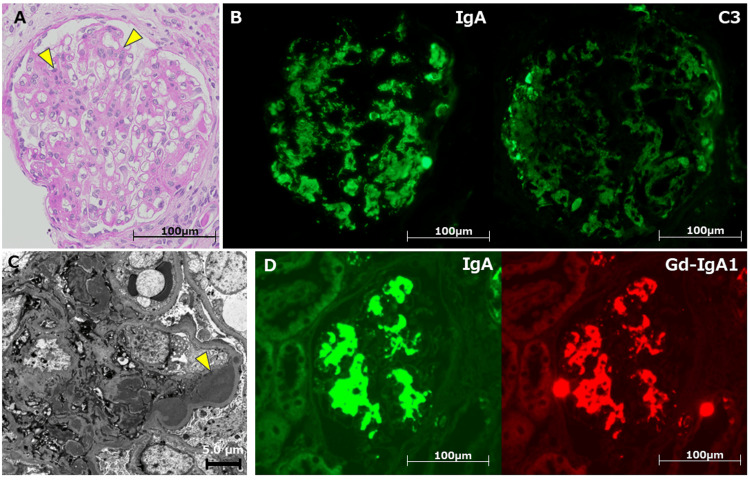
Renal biopsy confirmed the diagnosis of IgAV A: Light microscopy showed diffuse mesangial proliferative glomerulonephritis without any crescent formation. Arrowheads indicate mesangial proliferation (H&E staining). B: The immunofluorescence analysis indicated mesangial deposition of IgA and C3c. C: Electron microscopic findings confirmed electron-dense deposits in the mesangial area. D: Gd-IgA1 colocalized with IgA in the mesangial region using an anti-Gd-IgA1 monoclonal antibody. IgAV: IgA vasculitis, H&E: hematoxylin and eosin, IgA: immunoglobulin A

On day 6, a renal biopsy was performed, demonstrating mesangial proliferative glomerulonephritis (Figure 4A). The immunofluorescence analysis showed mesangial deposition of IgA and C3c (Figure 4B). Electron microscopy indicated electron-dense deposits in mesangium (Figure 4C). Furthermore, we confirmed colocalization of aberrantly glycosylated IgA1 (Gd-IgA1) with IgA in the mesangial region using an anti-Gd-IgA1 monoclonal antibody (Figure 4D).

**Figure 5:**
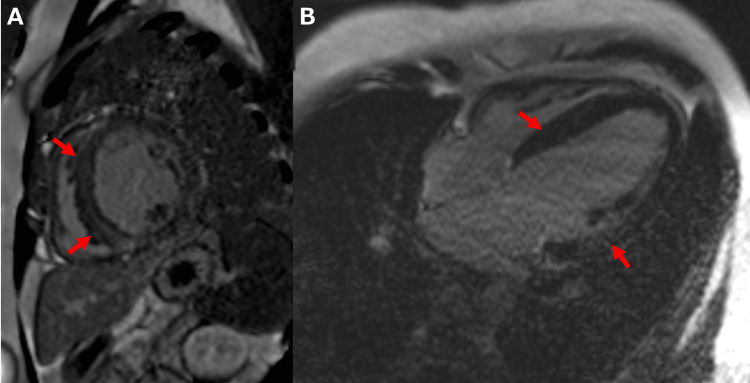
CMR imaging with late gadolinium enhancement Short-axis view (A) and four-chamber view (B) show patchy and linear abnormal enhancement (arrows) at the right ventricular junction of the ventricular septum and in the left ventricular mid-myocardium. CMR: cardiac magnetic resonance

Contrast-enhanced cardiac magnetic resonance (CMR) revealed increased left ventricular myocardial weight (75.9 g/m2), diffusely reduced left ventricular wall motion, and highly decreased LVEF (18.3%). Late gadolinium enhancement showed patchy and linear abnormal enhancement at the right ventricular junction of the ventricular septum and in the left ventricular mid-myocardium, a pattern seen in hypertensive heart disease (Figure 5A-5B).

**Figure 6:**
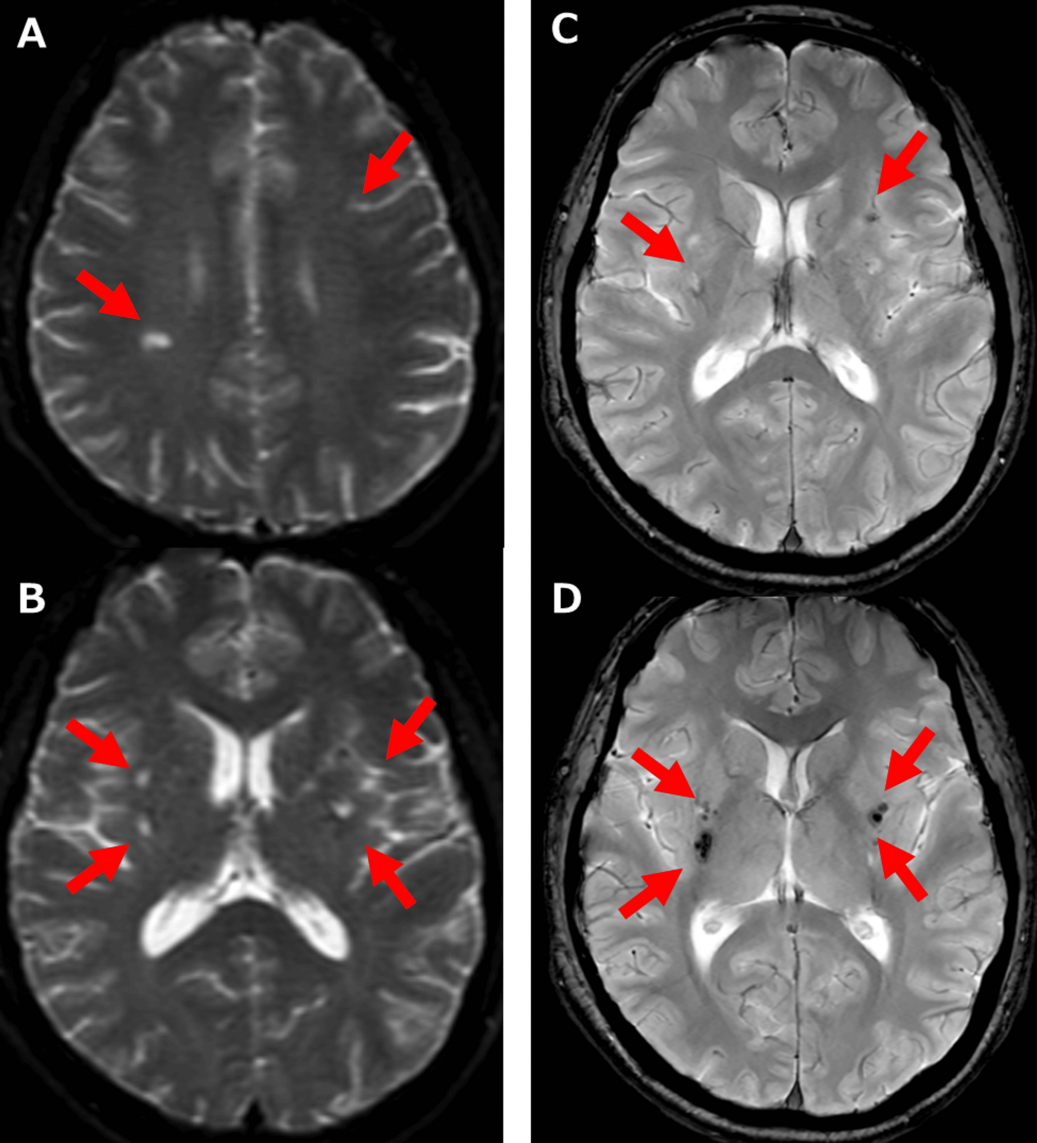
Brain MRI A, B: Diffusion-weighted images showing multiple increased signal intensities (arrows), indicating multiple cerebral infarctions. C, D: T2 star-weighted images revealing multiple decreased-signal intensities in the basal ganglia (arrows), demonstrating multiple microbleeds. MRI: magnetic resonance imaging

Although asymptomatic, brain MRI showed multiple increased signal intensities on diffusion-weighted images, indicating multiple cerebral infarctions (Figure 6A-6B). It also showed multiple decreased signal intensities in the basal ganglia on T2 star-weighted images, indicating multiple micro-bleeds (Figure 6C-6D).

**Figure 7:**
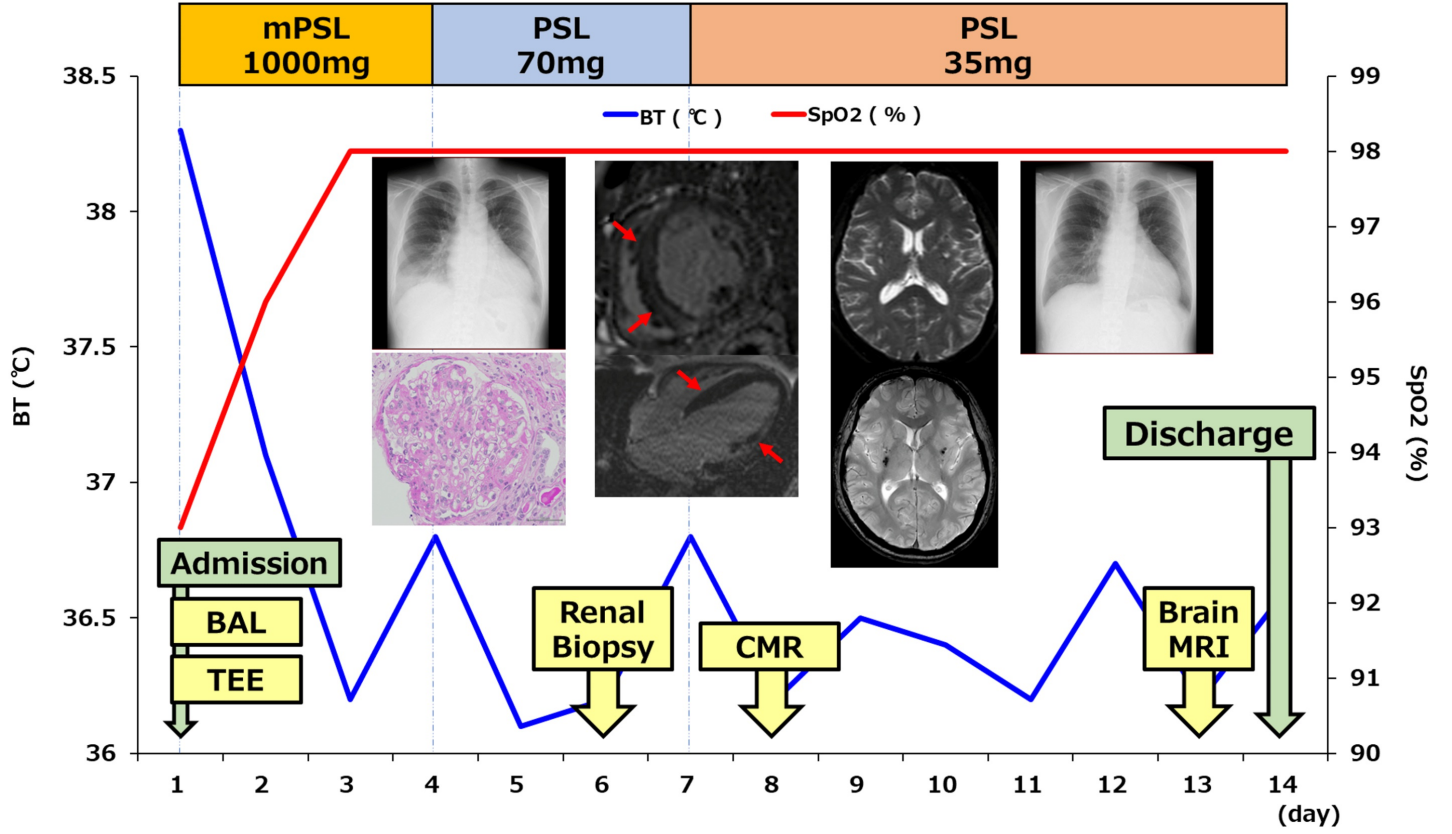
Summary of the patient's clinical course mPSL: methylprednisolone, PSL: prednisolone, BT: body temperature, BAL: bronchoalveolar lavage, TEE: transthoracic echocardiogram, CMR: cardiac magnetic resonance, MRI: magnetic resonance imaging

IgAV seemed to be the most likely explanation for these findings. As renal histological findings showed relatively mild renal injuries and the patient's airway symptoms improved, the PSL was reduced to 35 mg (0.5 mg/kg/day) on day 7 after consultation with the nephrologist. The patient was discharged on day 14 when his condition stabilized. The alveolar hemorrhage resolved, and the ejection fraction in the TTE improved to 53%. The patient is now primarily followed up in the nephrology outpatient clinic. His clinical course is illustrated in Figure 7.

## Discussion

The patient initially presented with DAH, accompanied by renal dysfunction, heart failure, multiple cerebral infarctions, and multiple cerebral microbleeds. While these multiple complications might suggest concurrent pathologies, several features strongly support a unified diagnosis of IgAV. Although the typical tetrad was not fully observed initially, the patient exhibited two key diagnostic components: purpura and nephritis. Though initially overlooked by the patient, the purpura on the lower extremities is consistent with adult IgAV cases, where skin manifestations may be less prominent [6]. The diagnostic process was systematic and comprehensive. While ANCA-associated vasculitis was initially suspected, autoimmune markers were negative. Although the skin biopsy showed inflammatory changes without typical vasculitic findings or IgA deposition, this does not exclude IgAV, as these findings can vary depending on biopsy timing and location [7,8]. The renal involvement, evident through dysfunction and later confirmed by biopsy showing IgA nephropathy, provided crucial diagnostic evidence. The diagnosis of IgAV was further strengthened by several factors: first, the combination of purpura and nephritis, along with the absence of other explainable causes of vasculitis [9,10]. Second, the rapid improvement with corticosteroid therapy strongly suggested an immune-mediated process, particularly in both pulmonary hemorrhage and cardiac function. Third, the simultaneous occurrence of multiple organ involvement in a previously healthy young adult indicated underlying systemic vasculitis rather than isolated pathologies. Additional support came from advanced pathological analysis using an anti-Gd-IgA1 monoclonal antibody, which revealed Gd-IgA1 colocalizing with IgA in the mesangial region. This finding aligns with the IgAV diagnosis and suggests shared pathogenic features with primary IgA nephropathy [11]. This case exemplifies the importance of recognizing that IgAV can manifest without classic signs, particularly when complicated by pulmonary-renal syndrome [3,12-15]. The severity of complications in our case is noteworthy: alveolar hemorrhage, occurring in only 0.8% to 5.0% of cases, is associated with a 27.6% mortality rate and is more common in older male patients [3]. Cardiovascular complications are extremely rare and predominantly seen in adult patients [4], while stroke remains an exceptionally uncommon neurologic complication in severe IgAV [5,16]. Based on (1) the favorable response to corticosteroid therapy, (2) the pathological findings of Gd-IgA1, (3) the simultaneity of multiple organ involvement, and (4) the absence of findings suggesting other vasculitis, this case is most appropriately considered as IgAV presenting with an extremely rare combination of complications.

## Conclusions

The dramatic response to corticosteroid therapy, along with the pathological evidence of Gd-IgA1 deposition and the pattern of multiple organ involvement in this young patient, strongly support systemic IgAV as the underlying pathological process rather than separate organ-specific pathologies. The simultaneous presentation of DAH (mortality rate 27.6%), heart failure, and stroke - three extremely rare complications in IgAV - makes this case particularly noteworthy.

To the best of our knowledge, there have been no reports of cases with these three overlapping serious complications, as seen in the present case. This case emphasizes that in adult-onset pulmonary-renal syndrome with additional complications such as heart failure or stroke. In contrast, various vasculitis syndromes are typically suspected, and IgAV should be considered a potential differential diagnosis, even in the absence of the typical tetrad. Early recognition and prompt initiation of appropriate therapy may be crucial for favorable outcomes in such cases with multiple severe complications.
